# Incidence and risk factors of hypertriglyceridemia in males with human immunodeficiency virus who are treated with combination antiretroviral therapy: a retrospective cohort study

**DOI:** 10.1186/s12944-023-01786-3

**Published:** 2023-02-23

**Authors:** Xian-dong Yu, Huihuang Huang, Yanmei Jiao, Jing Li, Xing Fan, Dawei Zhang, Fu-sheng Wang

**Affiliations:** 1 School of Public Health, Bengbu Medical College, Bengbu, 233000 Anhui China; 2 Senior Department of Infectious Diseases, The Fifth Medical Center of Chinese PLA General Hospital, Beijing, 100039 China; 3 Peking University 302 Clinical Medical School, Beijing, 100039 China

**Keywords:** HIV, ART, Hypertriglyceridemia, Incidence, Risk factor

## Abstract

**Background:**

Hypertriglyceridemia is associated with subclinical atherosclerosis and vascular inflammation even when low-density lipoprotein cholesterol levels are normal. However, few cohort studies on hypertriglyceridemia have been conducted in males with higher susceptibility to human immunodeficiency virus (HIV)-related deterioration of arterial structure and function. Our objective was to investigate the incidence of hypertriglyceridemia during treatment with combination antiretroviral therapy (cART) in males with HIV and explore its related risk factors.

**Methods:**

In this retrospective study, we included 309 males living with HIV (median age 31 years [interquartile range 26–42.5]) who initiated cART treatment in our hospital from January 2013 to December 2018. We collected follow-up data on serum triglycerides and other related information as of June 31, 2021. A Cox proportional hazards regression model was used to analyze the related risk factors.

**Results:**

In 666.7 person-years, hypertriglyceridemia occurred in 140 patients (triglyceride ≥2.3 mmol/L [200 mg/dL]), and the incidence rate was 21.0 per 100 person-years (Patients who took the lamivudine [3TC] + tenofovir disoproxil fumarate [TDF] + efavirenz [EFV] regimen accounted for 77.0% of the total patients.). Multiple Cox regression analysis showed that baseline CD4/CD8 ratio < 0.20 (hazard ratio [HR], 2.705 [95% confidence interval (CI): 1.381–5.296]; *P* = 0.004}, body mass index (BMI) ≥ 24.0 kg/m^2^ (HR, 1.768 [95% CI: 1.225–2.552]; *P* = 0.002), borderline high triglyceride at baseline (HR, 3.457 [95% CI: 2.162–5.527]; *P* < 0.001), and 3TC + zidovudine (AZT) + EFV regimen (HR, 2.702 [95% CI: 1.593–4.581]; *P* < 0.001), or 3TC + TDF + lopinavir/ritonavir (LPV/r) regimen (HR, 4.349 [95% CI: 2.664–7.102]; *P* < 0.001) were independent risk factors for hypertriglyceridemia.

**Conclusion:**

During the course of cART treatment, the incidence of hypertriglyceridemia in males with HIV was high. The main risk factors influencing its occurrence are a low baseline CD4/CD8 ratio, overweight and obesity, and the use of AZT or LPV/r in the cART regimen.

## Background

With the advent of combination antiretroviral therapy (cART) with high antiviral efficiency, human immunodeficiency virus (HIV) infection has changed from a fatal to a chronic disorder, but the clinical use of cART has been associated with a wide range of metabolic disorders [1, 2]. Furthermore, since cART regimens have led to a noteworthy extension of life expectancy in people living with HIV, prolonged lipid and glucose metabolism imbalances could significantly affect the long-term prognosis and outcome.

Low-level inflammation and chronic immune activation act on lipids through various mechanisms, making them more prone to atherosclerosis [2]. Heightened inflammation, residual HIV infection, and highly atherogenic lipoproteins contribute to a specific type of atherosclerosis in HIV, characterized by a higher prevalence of rupture-prone coronary plaques than that of ‘ordinary’ atherosclerosis [3]. Sun D’s al. found that HIV infection resulted in worse vascular status in all age groups and that men were more likely to undergo HIV-related deterioration in arterial structure and function [4]. There is an increasing concern, particularly regarding the elevated risk of cardiovascular events and acute pancreatitis [1, 5]. Hypertriglyceridemia is the most common dyslipidemia in people living with HIV, whether or not they have been treated with cART [1, 2, 6]. The effect of cART on lipid levels varies greatly among classes of cART drugs, and even among drugs within the same class. Generally, protease inhibitors, nucleoside reverse-transcriptase inhibitors (NRTIs), and non-NRTIs (NNRTIs) all increase triglyceride levels and newer drugs have more favorable effects compared to older ones [1, 2]. In addition, studies found that the prevalence of hypertriglyceridemia was higher in males with HIV [7, 8]. A recent study showed that hypertriglyceridemia is associated with subclinical atherosclerosis and vascular inflammation even in participants with normal low-density lipoprotein cholesterol levels [9]. Although a hypolipidemic diet and physical exercise may certainly improve dyslipidemia, pharmacological therapy becomes indispensable when plasma lipid levels are excessively increased or persist for a long time. However, prevention of related events is particularly important because of intolerance, potential drug interactions, and decreased patient adherence to multiple pharmacological regimens. Understanding the incidence of hypertriglyceridemia and its risk factors during cART will better meet the health needs of this population. Currently, only a few related cohort studies have been conducted. This study retrospectively analyzed the follow-up data of males living with HIV receiving cART enrolled from 2013 to 2018 to provide the basis for the early identification of hypertriglyceridemia and the rational choice of cART regimen for Chinese males with HIV during antiviral treatment.

## Methods

### Study population

This was a retrospective cohort study of people living with HIV who initiated cART treatment at the Fifth Medical Center of PLA General Hospital from January 1, 2013 to December 31, 2018. Inclusion criteria were: (1) individuals aged 18 years or older; (2) males living with HIV; (3) relevant clinical data such as baseline serum triglyceride (TG) levels, baseline HIV ribonucleic acid (RNA) level, and baseline CD4^+^ T lymphocyte count; and (4) the time interval from diagnosis to initial treatment was < 12 months. Exclusion criteria were: (1) patients who were taking lipid-lowering drugs; (2) baseline serum TG ≥ 2.3 mmol/L (200 mg/dL); (3) patients who were treated with cART before follow-up; and (4) those with tumors, autoimmune disease, kidney failure, and liver cirrhosis.

For eligible participants, serum TG levels and other relevant clinical data were collected from patients up to June 31, 2021. During the first 3 months after the start of cART, patients were followed up approximately once a month and then every 3 months (the specific time was based on the actual visit time of the patient). A patient was considered lost to follow-up if the interval between visits exceeded 12 months, or if the patient’s lipid data were missing for > 12 months.

The primary endpoint was hypertriglyceridemia (serum TG ≥ 2.3 mmol/L [200 mg/dL]). The occurrence of serum TG ≥ 2.3 mmol/L was considered a target event during the follow-up period (all participants were asked to fast overnight for approximately 12 h). For patients who switched to cART regimens during the follow-up, the first appearance of TG ≥ 2.3 mmol/L was taken as a node, and the cART regimen at that time was recorded. The relevant information of the participants included in the group mainly included demographic and clinical characteristics, and laboratory results. The demographic and clinical data collected included age, sex, body weight, height, mode of HIV acquisition, cART regimen, education level, time of HIV diagnosis, and presence of diabetes. Laboratory tests included TG (mmol/L), plasma glucose (mmol/L), total cholesterol (mmol/L), CD4 cell count (cells/μL), CD8 cell count (cells/μL), plasma HIV RNA (copies/mL), and alanine aminotransferase (ALT, U/L).

### Definitions and diagnostic criteria

According to current clinical practice guidelines for the treatment of dyslipidemia, it is recommended to start using statins when the serum TG > 2.3 mmol/L (200 mg/dL) [10]. Hypertriglyceridemia in our study was determined according to the Guidelines on National Cholesterol Education Program (NCEP) Adult Treatment Panel (ATP) III [11]. It was defined as serum TG ≥ 2.3 mmol/L at follow-up. In addition, TG ≥ 1.7 mmol/L, defined as borderline high TG, used in the classification of TG levels at baseline.

The diagnosis of HIV infection was made in accordance with the diagnostic criteria of the Chinese guidelines for the diagnosis and treatment of HIV/acquired immunodeficiency syndrome (AIDS) (2021 edition)” [12]. Body mass index (BMI) = measured body mass (kg) ÷ height (m^2^): BMI ≥ 24.0 kg/m^2^ was defined as being overweight or obese; 18.5–23.9 kg/m^2^ as normal; and <18.5 kg/m^2^ as being underweight [13]. Liver injury was diagnosed by referring to “EASL 2017 Clinical Practice Guidelines on the management of hepatitis B virus infection.” The occurrence of liver injury was defined as an ALT level 1.25 times higher than the upper limit of a normal standard value (ALT > 50 U/L, upper limit of normal [ULN] approximately 40 U/L) [14]. Hyperglycemia was diagnosed according to “Standards of Medical Care for Type 2 Diabetes in China 2019”: fasting plasma glucose (FPG) ≥ 6.1 mmol/L or under oral hypoglycemic drugs or insulin treatment [15].

### Statistical analysis

All analyses were performed using Statistical Package for the Social Sciences (SPSS) version 25.0 (IBM SPSS, Chicago, IL, USA) or GraphPad Prism 9. The Shapiro–Wilk test was used to test whether the data conformed to a normal distribution. When continuous variables were normally distributed, the mean and standard deviation (SD) were used. When continuous variables were non-normally distributed, the median with interquartile range (IQR) was used. Categorical variables were summarized as numbers (%).

As patients entered the cohort at different times, the incidence density was calculated to account for the occurrence of hypertriglyceridemia during follow-up. Person-years were counted on an individual basis, starting from the date cART treatment was initiated and ending with the time of hypertriglyceridemia detection, a follow-up period of 48 months, discontinuation of the cART regimen, last lipid test, or June 31, 2021, the final date of the follow-up, whichever occurred first. The incidence of hypertriglyceridemia was defined as the number of new cases during the follow-up period divided by the number of person-years of follow-up. The cumulative incidence and Kaplan–Meier curves of hypertriglyceridemia were drawn using GraphPad Prism 9, and the results of the log-rank test were calculated using GraphPad Prism 9. The approximate normal distribution method was used to calculate the 95% confidence interval (CI) for the incidence rate.

Cox proportional hazards models were used to analyze risk factors for hypertriglyceridemia. Variables with *P* < 0.20 in univariate analysis were entered into a multiple Cox proportional hazard model. All reported *P*-values were two-sided. Statistical significance was set at *P* < 0.05. The adverse effects of dyslipidemia on blood vessels are a relatively slow process. In the first few months of initiating cART for people living with HIV, the relevant indicators may fluctuate due to the sudden effects of antiviral drugs. To make the research results more practical, we excluded events within 3 months of the baseline.

## Results

**Fig. 1:**
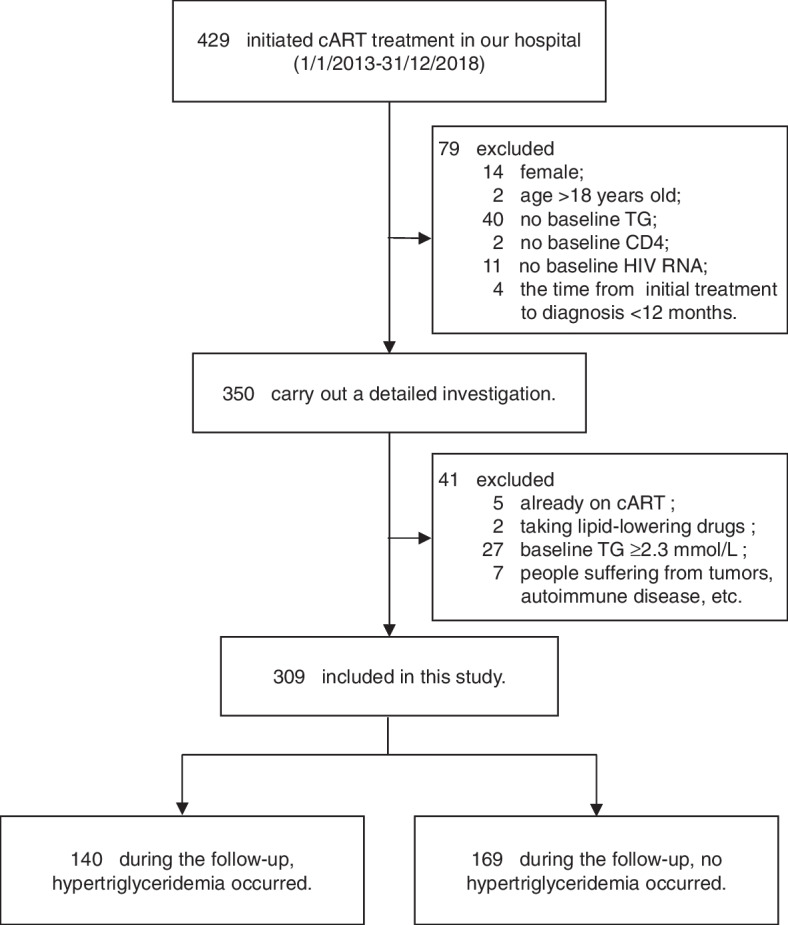
Flowchart of patient selection for the study

A total of 309 males with HIV met the study criteria from January 1, 2013 to June 31, 2018 (Fig. 1). They had a median age of 31 years (IQR, 26–42.5) and a median CD4 cell count of 293 cells/μL (IQR 192–418). The patient’s primary antiviral regimen was lamivudine (3TC) + tenofovir disoproxil fumarate (TDF) + efavirenz (EFV), which accounted for 77.0% of the total (Table 1).

**Table 1:** Baseline characteristics (*n* = 309, all male)

Characteristic	Value
Median (IQR) age, y	31 (26–42.5)
Median (IQR) BMI, kg/m^2 a^	22.15 (20.32–24.22)
Median (IQR) plasma glucose, mmol/L	5.30 (5.00–5.60)
Median (IQR) ALT, U/L	20 (14–28)
Median (IQR) CD4^+^ count, cells/μL	293 (192–418)
Median (IQR) CD8^+^ count, cells/μL	886 (628.5–1275)
Median (IQR) CD4/CD8 ratio	0.31 (0.20–0.45)
Geometric median (IQR) viral load, copies/mL ^b^	4.67 (4.16–5.21)
Mean (SD) TC, mmol/L	3.72 ± 0.89
Mode of HIV acquisition ^c^	
Hetero	48 (15.5%)
MSM	243 (79.9%)
Other	13 (4.2%)
Degree of education	
High school and below	126 (40.8%)
University and above	106 (34.3%)
No information	77 (24.9%)
TG borderline high	
No	280 (90.6%)
Yes	29 (9.4%)
cART regimen	
3TC + TDF + EFV	238 (77.0%)
3TC + AZT + EFV	22 (7.1%)
3TC + TDF + LPV/r	31 (10.0%)
Other regimens	18 (5.8%)

Other regimens: 3TC + AZT + LPV/r, 3TC + ABC + LPV/r, 3TC + AZT + NVP, 3TC + ABC + EFV, 3TC + TDF + NVP, etc.

*Abbreviations*: *IQR* Interquartile range, *BMI* Body mass index, *SD* Standard deviation, *ALT* Alanine aminotransferase, *TC* Total cholesterol, *MSM* Men who have sex with men, *TG* Triglyceride, *3TC* Lamivudine, *EFV* Efavirenz, *LPV/r* Lopinavir/ritonavir, *TDF* Tenofovir disoproxil fumarate, *NVP* Nevirapine, *AZT* Zidovudine, *ABC* Abacavir, *y* Years old

^a^*n* = 307; ^b^ *n* = 308; ^c^ *n* = 304

**Fig. 2:**
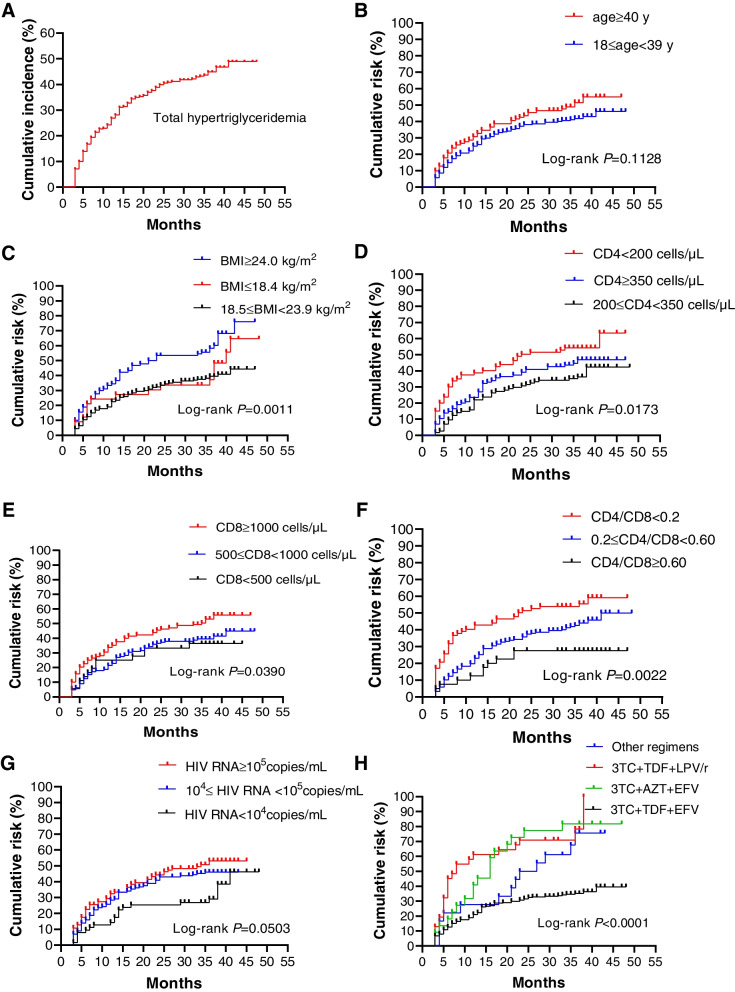
Cumulative incidence of hypertriglyceridemia during follow-up and Kaplan-Meier curves of its main risk factors. **A** Cumulative incidence of hypertriglyceridemia during follow-up; **B**-**H** Kaplan-Meier curve under different risk factors. Abbreviations: BMI, body mass index; 3TC, lamivudine; EFV, efavirenz; LPV/r, lopinavir/ritonavir; TDF, tenofovir disoproxil fumarate; AZT, Zidovudine; y, years old

Figure 2. A shows the cumulative incidence of hypertriglyceridemia during the follow-up period, and we can see that the curve gradually flattens out, indicating a gradual decline in the incidence of hypertriglyceridemia in our patient. Older participants showed a higher incidence of hypertriglyceridemia, but the differences were not statistically significant on the log-rank test (Fig. 2B). Both lower and higher BMI showed a higher risk of hypertriglyceridemia, and the subgroup with lower BMI appeared to have a higher incidence of hypertriglyceridemia at the beginning, although this might be related to the smaller sample size (23 cases, Table 2) of this subgroup (Fig. 2C). Low CD4 and CD8 cell count showed the opposite trend. Low CD4 cell count was associated with high risk of hypertriglyceridemia (Fig. 2D), while low CD8 cell count was associated with low incidence of hypertriglyceridemia (Fig. 2E), and low CD4/CD8 ratio showed the same trend as low CD4 cell count (Fig. 2F), notably the statistical differences between the relevant subgroups of CD4/CD8 ratio were more significant. Low HIV viral load indicated low risk, but there were no statistical differences between HIV RNA-associated subgroups (Fig. 2G). The antiviral regimens 3TC + TDF + lopinavir/ritonavir (LPV/r) and 3TC + azidothymidine (AZT) + EFV were significantly different from 3TC + TDF + EFV, with the 3TC + TDF + LPV/r regimen showing a significant short-term effect on hypertriglyceridemia and the effect of the 3TC + AZT + EFV regimen was stable compared with that of the 3TC + TDF + LPV/r regimen (Fig. 2H).

**Table 2:** Incidence of hypertriglyceridemia and univariate Cox regression analysis in each subgroup (*n* = 309, all male)

	Events/patients	PYFU	Incidence per /100 PYFU (95% ***CI***)	***HR*** (95%***CI)***	***P*** Value	Global ***P***
**Age (years)**						
18 ~ 39	88/208	462.8	19.0 (15.4, 22.6)	Ref		**0.119**
≥ 40	52/101	203.9	25.5 (19.5, 31.5)	1.313 (0.932–1.851)	0.119	
**BMI (kg/m**^**2**^**)** ^**a**^						
18.5 ~ 23.9	80/201	462.6	17.3 (13.8, 20.7)	Ref		**0.004**
≤ 18.4	9/23	49.9	18.0 (7.4, 28.7)	1.068 (0.536–2.129)	0.851	
≥ 24.0	50/83	151.3	33.1 (25.6, 40.6)	1.817 (1.275–2.589)	0.001	
**Mode of HIV acquisition** ^**b**^						
Hetero	22/48	98.1	22.4 (14.2, 30.7)	Ref		0.918
MSM	113/243	525.5	21.5 (18.0, 25.0)	0.956 (0.605–1.509)	0.845	
Other	5/13	27.9	17.9 (3.7, 32.1)	0.815 (0.309–2.154)	0.680	
**Degree of education** ^**c**^						
High school and below	57/126	266.3	21.4 (16.5, 26.3)	Ref		0.509
University and above	45/106	244.6	18.4 (13.5, 23.3)	0.876 (0.592–1.297)	0.509	
**CD4 cell count (cells/μL)**						
≥ 350	53/115	248.3	21.3 (16.2, 26.4)	Ref		0.021
200 ~ 349	43/114	270.0	15.9 (11.6, 20.3)	0.758 (0.507–1.134)	0.178	
< 200	44/80	148.3	29.7 (22.3, 37.0)	1.376 (0.922–2.052)	0.118	
**CD8 cell count (cells/μL)**						
≥ 1000	68/128	252.9	26.9 (21.4, 32.4)	Ref		0.045
500 ~ 999	59/145	330.7	17.8 (13.7, 22.0)	0.673 (0.475–0.954)	0.026	
< 500	13/36	83.1	15.6 (7.8, 23.5)	0.599 (0.331–1.084)	0.090	
**CD4/CD8 ratio**						
≥ 0.60	11/40	103.5	10.6 (4.7, 16.6)	Ref		**0.003**
0.20 ~ 0.59	83/187	417.1	19.9 (16.1, 23.7)	1.799 (0.958–3.377)	0.068	
< 0.20	46/82	146.1	31.5 (24.0, 39.0)	2.803 (1.449–5.422)	0.002	
**HIV RNA (log**_**10**_ **copies/mL)**						
< 4.00	21/63	158.9	13.2 (7.9, 18.5)	Ref		**0.058**
4.00 ~ 4.99	66/144	303.3	21.8 (17.1, 26.4)	1.562 (0.956–2.553)	0.075	
≥ 5.00	53/102	204.5	25.9 (19.9, 31.9)	1.850 (1.116–3.068)	0.017	
**ALT(U/L)**						
≤ 50 (1.25 ULN)	131/289	624.0	21.0 (17.8, 24.2)	Ref		0.963
> 50 (1.25 ULN)	9/20	42.7	21.1 (8.9, 33.3)	1.016 (0.517–1.997)	0.963	
**Plasma glucose (mmol/L)**						
< 6.1	119/267	577.9	20.6 (17.3, 23.9)	Ref		0.589
≥ 6.1	21/42	88.8	23.7 (14.8, 32.5)	1.136 (0.714–1.808)	0.589	
**TC (mmol/L)** ^**d**^						
< 5.2	134/294	633.7	21.1 (18.0, 24.3)	Ref		0.871
≥ 5.2	6/14	30.2	19.9 (5.6, 34.1)	0.934 (0.412–2.117)	0.871	
**TG borderline high**						
No	115/280	636.6	18.1 (15.1, 21.1)	Ref		**< 0.001**
Yes	25/29	30.1	83.1 (69.7, 96.5)	3.948 (2.546–6.122)	< 0.001	
**cART regimen**						
3TC + TDF + EFV	85/238	557.8	15.2 (12.3, 18.2)	Ref		**< 0.001**
3TC + AZT + EFV	18/22	33.9	53.1 (36.3, 69.9)	3.048 (1.827–5.084)	< 0.001	
3TC + TDF + LPV/r	24/31	39.4	60.9 (45.7, 76.1)	3.497 (2.213–5.527)	< 0.001	
Other regimens	13/18	35.6	36.5 (20.7, 52.4)	2.276 (1.269–4.083)	0.006	
**Overall**	**140/309**	**666.7**	**21.0 (17.9, 24.1)**			

*Abbreviations*: *PYFU* Person-years of follow-up, *ULN* Upper limit of normal

^a^*n* = 307; ^b^*n* = 304; ^c^*n* = 232; ^d^*n* = 308

The total number of person-years followed in this study was 666.7 (mean 2.16 years [range, 0.25–4.0]), during which 140 patients developed hypertriglyceridemia with an incidence of 21.0/100 person-years (Table 2). The incidence of hypertriglyceridemia was 19.9/100 and 10.6/100 person-years in patients with CD4/CD8 ratio between 0.20 and 0.59 and those with CD4/CD8 ratio > 0.60, respectively. Patients with a CD4/CD8 ratio < 0.20 had an incidence of 31.5/100 person-years. The incidence rate of patients with borderline high TG at baseline was 83.1/100 person-years. The incidence rates in other subgroups are shown in Table 2. Univariate Cox regression analysis showed that BMI ≥ 24.0 kg/m^2^(*P* = 0.001), CD8 cell count ≥1000 cells/μL (*P* = 0.045), HIV RNA load ≥5 log_10_ copies/mL (*P* = 0.017), borderline high TG at baseline (*P* < 0.001), 3TC + AZT + EFV (*P* < 0.001), and 3TC + TDF + LPV/r (*P* < 0.001) regimen were risk factors for hypertriglyceridemia in patients receiving cART treatment. Age, mode of HIV acquisition, education, ALT, plasma glucose, and TC levels were not significantly associated with hypertriglyceridemia in the univariate analysis (Table 2).

**Fig. 3:**
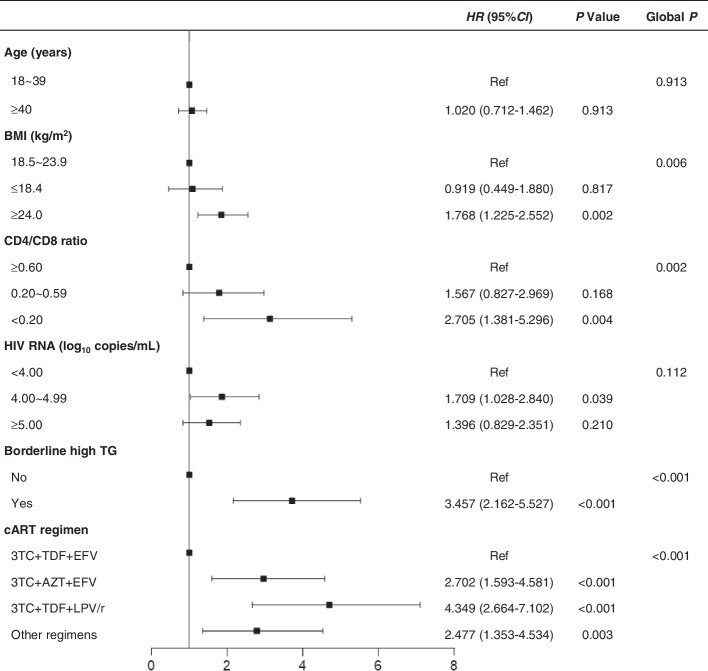
Multiple Cox regression analysis of risk factors associated with hypertriglyceridemia in males with HIV receiving cART. CD4 cell count, CD8 cell count, and CD4/CD8 ratio were collinear, and only the index with the smallest *P* value was included in the multivariate analysis. Other regimens: 3TC + AZT + LPV/r, 3TC + ABC + LPV/r, 3TC + AZT + NVP, 3TC + ABC + EFV, 3TC + TDF + NVP, etc

Variables in the multiple Cox models included age, BMI, CD4/CD8 ratio, HIV RNA, borderline high TG, and cART regimen. The results showed that BMI, CD4/CD8 ratio, borderline high TG, and cART regimen were independently associated with the occurrence of hypertriglyceridemia (Fig. 3). Within subsets of each independent risk factor, BMI ≥ 24.0 kg/m^2^ (hazard ratio [HR], 1.768 (95% CI: 1.225–2.552]; *P* = 0.002), CD4/CD8 ratio < 0.20 (HR, 2.705 [95% CI: 1.381–5.296]; *P* = 0.004), borderline high TG (HR, 3.457 [95% CI: 2.162–5.527]; *P* < 0.001), 3TC + AZT + EFV regimen (HR, 2.702 [95% CI: 1.593–4.581]; *P* < 0.001), or 3TC + TDF + LPV/r regimen (HR, 4.349 [95% CI: 2.664–7.102]; *P* < 0.001) were statistically significant (Fig. 3). While a subset of HIV RNA was statistically significant, the overall difference in this item was not statistically significant (Fig. 3). Age was not significantly correlated with the occurrence of hypertriglyceridemia.

## Discussion

In this study, the incidence of hypertriglyceridemia during antiretroviral treatment was 21.0/100 person-years among males with HIV who were treated early with cART upon diagnosis. Multiple Cox regression analysis showed that BMI ≥ 24.0 kg/m^2^, baseline CD4/CD8 ratio < 0.20, borderline high TG at baseline, and the use of the 3TC + AZT + EFV regimen or 3TC + TDF + LPV/r regimen were independent risk factors for hypertriglyceridemia.

In a cohort of 4577 persons living with HIV, the incidence of dyslipidemia (dyslipidemia was defined as random TC > 240 mg/dL, high density lipoprotein [HDL] < 35 mg/dL, TG > 200 mg/dL, or initiation of lipid-lowering therapy) during antiretroviral treatment in cART-naive people was 227.7/1000 person-years [16]. One possible reason the incidence of hypertriglyceridemia was lower than that in our study was that the study included both female and Black individuals. Studies have found that the incidence of dyslipidemia is significantly lower in female and Black ethnicity [16, 17]. However, our study only included Chinese males. Other reasons include different cART regimens and economic levels. This also demonstrates that the incidence of hypertriglyceridemia in males living with HIV is high.

Multivariate analysis showed that the incidence of hypertriglyceridemia in overweight or obese patients was higher than in those with normal weight. This suggests that overweight or obese patients should pay more attention to cART regimen selection. Simultaneously, they should pay attention to changing their lifestyle and reducing their weight to the normal range as soon as possible. The presence of CD4 cell counts < 200 cells/μL before treatment in people living with HIV usually indicates that the patient’s immunity has been severely damaged. At this time, even if patients with CD4 cell counts < 200 cells/μL receive the same cART regimen as those with CD4 cell counts > 350 cells/μL, patients’ immunity recovery is weak, never reaching the level achieved by patients with CD4 cell counts > 350 cells/μL. Grunfeld C et al. found that TG levels were above normal in patients with CD4 cell counts < 200 and approximately twice normal in symptomatic patients with AIDS [18]. In our study, we found that both CD4 < 200 cells/μL and CD8 ≥ 1000 cells/μL were at high risk for hypertriglyceridemia, and the difference was more significant when expressed as the CD4/CD8 ratio. Multivariate analysis also showed that baseline CD4/CD8 ratio < 0.20 was a risk factor for hypertriglyceridemia. This indicates that the serum TG level of the patients may be related to their immune status. The worse the immune function is, the more likely the TG level will increase. The possible reason is that the worse the immunity, the more susceptible it is to infection by other viruses, resulting in an increase in the secretion of interferon, which in turn leads to an increase in the level of TG [18, 19]. Low CD4 cell counts (< 200 cells/μL) have also been shown to be a risk factor for hypertriglyceridemia by Duan Y et al.’s study [20]. This suggests that people living with HIV must receive antiviral treatment as soon as possible to avoid irreversible damage to immunity caused by the virus and possibly a higher incidence of hypertriglyceridemia.

The HIV viral load is associated with the disease stage in patients and is relatively high in those who have just been infected or are in the AIDS phase. Statistically significant differences in the incidence of hypertriglyceridemia between the subgroup with geometric viral loads between 4.00 and 4.99 and the subgroup with geometric viral loads < 4.00 may reflect differences in the patient’s immunity. Patients with geometric viral loads < 4.00 had greater immunity. Because our inclusion criteria required < 12 months from the initial diagnosis to treatment, the time to infection for most patients may be short, and the virus causes less damage to the patient’s immunity. This may explain the small difference between the subgroup with a geometric viral load > 5.00 and the subgroup with a geometric viral load < 4.00. In combination with the associated CD4/CD8 ratio results, the high risk of hypertriglyceridemia may be associated with the low baseline immunity in our patient. The patient’s low immunity may lead to an increase in susceptibility to related inflammation and infection and eventually to an increase in TG levels [21].

Our study showed that the risk of hypertriglyceridemia in patients with borderline high TG was 3.457 times higher than that in those with normal TG levels. For these patients, special lipid-lowering measures, such as strengthening physical exercise or targeted dietary changes, should be carried out at the same time as choosing the cART regimen. Ozemek et al. showed that physical activity and sports training could improve the cardiovascular health of adults living with HIV, and even in the presence of cardiovascular disease, it can help reduce the mortality rate [22]. Among the antiviral treatment regimens, there was a statistically significant difference in the incidence between 3TC + TDF + EFV and 3TC + TDF + LPV/r, and 3TC + AZT + EFV. Compared with 3TC + TDF + EFV, the two regimens have differences in AZT and LPV/r, which are consistent with the study results of Feeney ER [23] and Calza L [24] in that the inclusion of AZT or LPV/r in antiviral regimens may cause elevated serum TG levels in people living with HIV. Considering that 58.1% (not shown in this article) of patients on the 3TC + TDF + LPV/r regimen switched from other regimens during follow-up and that 22.7% of the patients on the 3TC + AZT + EFV regimen switched from other regimens during follow-up, the effect of the 3TC + TDF + LPV/r regimen on TG levels was likely to be stronger than that shown in Fig. 2. B. The strong influence of the antiviral regimen containing LPV/r on serum TG levels in our patients may be due to its inhibition of the nuclear form of sterol regulatory element binding proteins (regulating the level of fat and cholesterol in cells and plasma) degradation in the liver and adipose tissue nuclei or suppression of proteasome-mediated apolipoprotein B decomposition in the liver [25]. The effect of AZT on serum TG in patients indicated that AZT might have a chronic lesion on TG regulation in the body, just as it produces other toxic side effects [26].

The advantages of our study lie in its longitudinal cohort design, which is more reliable than that of the cross-sectional design in confirming causality, and it is of great theoretical and practical significance to guide clinical practice by analyzing related risk factors from a clinical perspective. However, there are also limitations, such as only male participants and single centers containing only people living with HIV-1 were included. In the future, females can be included in the study, and the sample size can be expanded to verify further and promote the conclusion of this study.

## Conclusions

In conclusion, the incidence of hypertriglyceridemia was high in male cART-naive participants during antiviral treatment. Therefore, clinicians should pay attention to the changes in TG levels of patients with baseline CD4/CD8 ratio < 0.20, BMI ≥ 24.0 kg/m^2^, or borderline high TG at baseline during antiviral treatment, and pay special attention to patients who use the antiviral regimen, including AZT or LPV/r, and check their TG levels regularly.

## Abbreviations

AIDS: Acquired immune deficiency syndrome
ABC: Abacavir
ALT: Alanine aminotransferase
AZT: Zidovudine
ART: Antiretroviral therapy
BMI: Body mass index
cART: Combination antiretroviral therapy
EFV: Efavirenz
HIV: Human immunodeficiency virus
IQR: Interquartile range
LDL-C: Low-density lipoprotein cholesterol
LPV/r: Lopinavir/ritonavir
MSM: Men who have sex with men
NRTIs: Nucleoside reverse-transcriptase inhibitors
NNRTIs: Non- nucleoside reverse-transcriptase inhibitors
NVP: Nevirapine
PYFU: Person-years of follow-up
ULN: Upper limit of normal.
SD: Standard deviation
TC: Total cholesterol
TG: Triglycerides
TDF: Tenofovir disoproxil fumarate
3TC: Lamivudine
y: Years old
